# Development of visual perception of others’ actions: Children’s judgment of lifted weight

**DOI:** 10.1371/journal.pone.0224979

**Published:** 2019-11-15

**Authors:** Alessandra Sciutti, Laura Patanè, Giulio Sandini

**Affiliations:** 1 Cognitive Architecture for Collaborative Technologies (CONTACT) Unit, Istituto Italiano di Tecnologia, Genova, Italy; 2 Robotics, Brain and Cognitive Sciences Department, Istituto Italiano di Tecnologia, Genova, Italy; University of Bologna, ITALY

## Abstract

Humans are excellent at perceiving different features of the actions performed by others. For instance, by viewing someone else manipulating an unknown object, one can infer its weight–an intrinsic feature otherwise not directly accessible through vision. How such perceptual skill develops during childhood remains unclear. To confront this gap, the current study had children (N:63, 6–10 years old) and adults (N:21) judge the weight of objects after observing videos of an actor lifting them. Although 6-year-olds could already discriminate different weights, judgment accuracy had not reached adult-like levels by 10 years of age. Additionally, children’s stature was a more reliable predictor of their ability to read others’ actions than was their chronological age. This paper discusses the results in light of a potential link between motor development and action perception.

## Introduction

Humans have a remarkable ability to understand different aspects of actions performed by others from mere observation. For instance, one can perceive subtle differences in the kinematic features of someone else’s reaching and use them to disambiguate from a number of potential goals [1]. Indeed, one can detect the intention to compete or cooperate in grasping an object simply by observing the initial movement phase [2], or alternatively infer whether someone is trying to deceive with a motion [3]. Even when the intention is clear, such as in the case of competitive sports, one can read others’ action properties to anticipate what is going to happen. Expert football players can infer where a kicker will direct the ball from the kicker’s initial body movements [4], while professional basketball players can predict success or failure of free shots at a basket from the very early phases of the throwing action [5]. In everyday life, humans are particularly skilled at detecting different aspects of others’ kinematics, which they use to reveal hidden properties of a manipulated object, and its mass in particular [6–9]. For example, if one observes someone else fetching a carton of milk from the refrigerator, one will easily understand whether it is full or empty and be prepared to handle when that someone passes it on.

In early development, infants already show sensitivity to some properties of others’ actions, as their goal [10,11], whether they have been successful or not [12], and whether there is an obstacle to goal achievement [13]. In infants just as in adults, the ability to read action properties goes beyond understanding others’ intentions [14,15]. For example, at 14 months of age, infants already show a differential electroencephalographic response to others reaching, grasping and lifting objects of different mass, so long as they have direct experience of the different weights and of their association with object color. This shows their sensitivity to different motor requirements associated with the same goal [16]. Older children (4 years old) can sort groups of identical objects into two categories based on weight, so long as they first observe a demonstrator sort similar objects according to this criterion [17,18]. More generally, in early childhood the ability to distinguish very heavy from very light objects by observing others is already developed, as indicated by the common joke in which children try to fool others by lifting an empty parcel as if it were very heavy. Although pretending to lift a heavy weight when it is actually light is not an easy task[19], when a child executes the trick well, the surprise of the companion when he lifts the same object proves he was processing the observed action to guess object weight. Kaiser and Profitt found that young children (five to seven years of age) can distinguish between wooden boxes of very different weights (7.27Kg, 25.45kg and 39.09kg) by looking at an actor lifting and transporting them [20]. In adulthood, reading weight information embedded in others’ actions becomes significantly more refined, where adults can reliably distinguish multiple object masses differing as little as 100g simply by observing someone else lifting them [7,21].

In the current study, we wanted to evaluate how this type of detailed action reading skill develops from a relatively crude distinction between heavy and light weight in early childhood to the ability of detecting subtle differences in action kinematics when lifting slightly different weights in adulthood. To investigate the development of this ability, we showed several clips of an adult woman lifting a series of identical bottles varying in weight from 100g to 400g with 100g steps to children (aged 6 to 10 years) and to a group of adults; this is a range of masses commonly manipulated even by the youngest group of children tested (6 years of age. We considered 6 year-olds for the younger group because it is when children start to distinguish among masses differing just 50g after lifting them and sort them correctly [22]. The weight estimates were then analyzed to assess the development in their action perception skills, both in terms of *accuracy*–the ability to attribute to each movement to the weight actually lifted–and *precision–*the variability of repeated estimates around the same lifted weight.

## Methods

### Subjects

84 healthy subjects recruited from a local elementary school and the local university agreed to voluntarily participate in the current study. Children came primarily from working-class and middle-class Caucasian families from a small neighborhood of a northern Italian city. Adults came from the same region and class. Among the youngest group (6-year-olds), three children could not understand the instructions of the experiment, and so the study excluded them from the sample. We executed data collection from nineteen 6-year-olds (9 females, 10 males, age: *M* = 6, *SD* = 0.5 years), nineteen 8-year-olds (10 females, 9 males, *M* = 8, *SD* = 0.3 years), twenty-two 10-year-olds (17 females, 5 males, *M* = 10, *SD* = 0.3 years) and twenty-one adults (14 females, 7 males, *M* = 32, *SD* = 6 years). All participants (or parents or guardians in the case of minors) gave written informed consent prior to testing, and the local ethics committee approved the study (Azienda Sanitaria Locale Genovese N.3).

### Procedure

**Fig 1:**
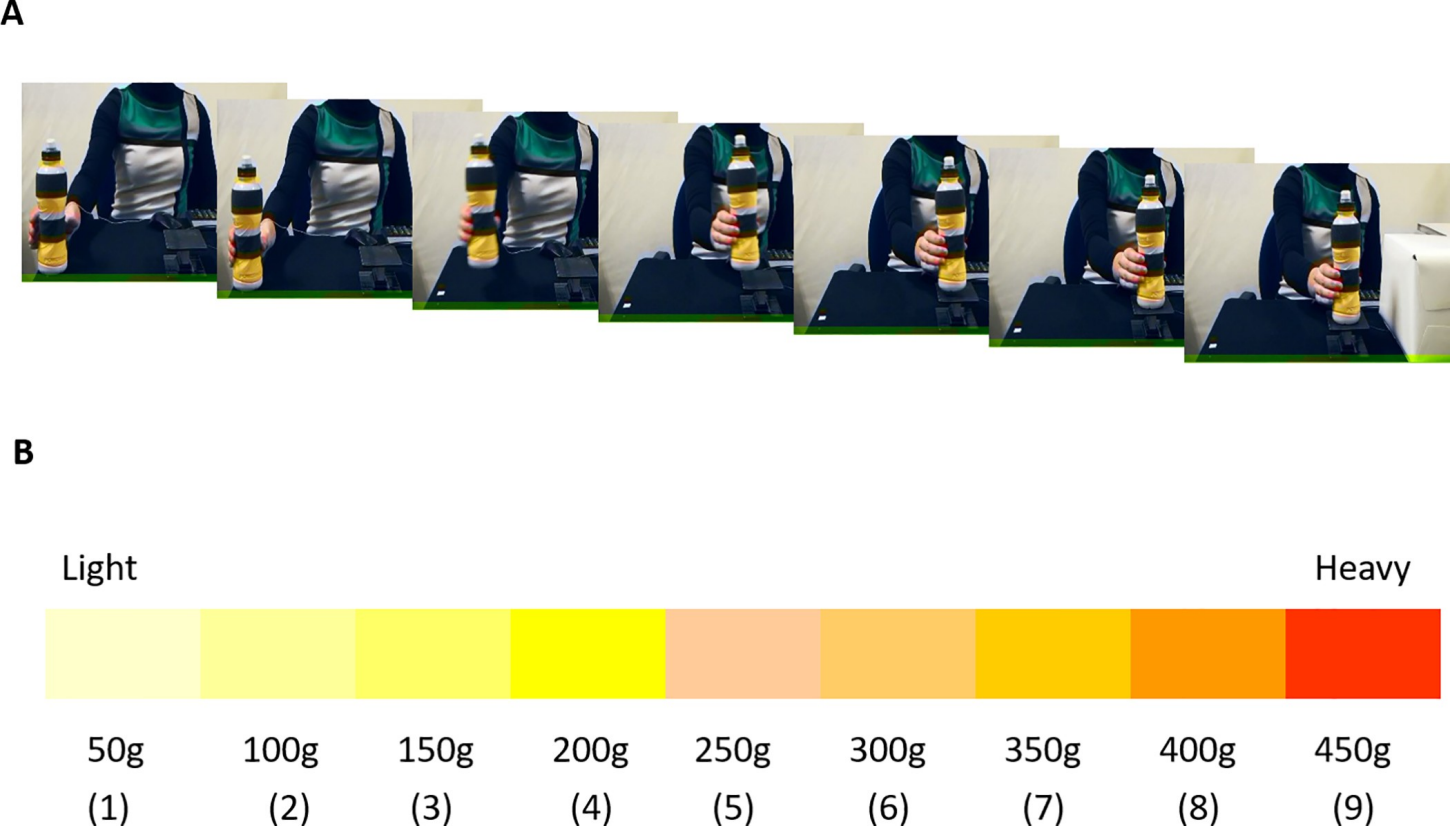
Stimuli. **A)** Still images from one of the clips used as stimulus, showing the actor grasping, lifting and placing a bottle on a raised platform. The bottle was 23cm tall, with a base of 6cm diameter and was opaque, to hide the amount of corn flour that determined its weight. **B)** Colored bar used by children to indicate which weight was lifted in the movie. The figure is similar but not identical to the original image, which contained also small drawings showing a person lifting a light or heavy load at the two sides of the bar. Therefore it is for illustrative purposes only.

Before experiment initiation, subjects lifted 6 bottles of known weight (50g, 100g, 200g, 300g, 400g and 450g) to experience the different loads, while the experimenter announced the corresponding weights. To simplify the task for the younger children, the current study employed a bar that was differently colored for each different weight, in addition to a figurative explanation of “light” or “heavy” (Fig 1B) to indicate the weight. Participants watched a set of movies where a human actor lifted various bottles, ranging from 100 to 400g with 100g steps (Fig 1A for a sample clip). Each bottle was 23cm tall and had a base of 6cm diameter. Each was also opaque to hide the amount of corn flour determining the weight of each bottle. After each movie, the study asked participants to estimate the weight of the bottle by selecting a number between 1 and 9, corresponding to a range from 50g to 450g with 50g increments. For children, the study used the colored bar to collect the answers and the experimenter entered each response (in the form of a number between 1 and 9) into the computer. The experimental session where participants had to judge the weight from lifting observation consisted of 20 trials (5 presentations for each weight) for children and 32 trials (8 presentations for each weight) for adults. We decided to opt for a smaller number of presentations for children to mitigate the risk of fatigue or loss of attention in case of a longer exercise, especially for the youngest group. The study randomized the order of presentation of the different weights. As a measure of physical development, height was measured for all participants.

### Setup and stimuli

Each participant sat in a comfortable chair in front of a 17-inch monitor (resolution, 1280x960 pixels) that displayed video clips with a custom routine. The routine was written in MATLAB using the Psychophysics Toolbox extensions [23,24]. In each movie, an actor grasped, lifted, transported and placed a set of bottles on a raised support. The bottles were apparently identical but of different weights (Fig 1A). The actor was a 29-year-old woman (height = 1.67m, mass = 60Kg). Each movie started with the hand of the actor behind the bottle about to be grasped. The videos lasted 3.39s (*SD =* 0.08s) on average. The demonstrator was a trained experimenter who had been informed of object weight before action initiation. This choice was made to replicate a real-life situation, wherein who manipulates the object is often aware of its weight, while the observer may not be. An Optotrak Certus system (frame rate: 250 Hz) recorded the actor’s movements by positioning an active marker on the back of her right hand. Table 1 presents the detailed properties of the presented actions. Repeated Measures ANOVAs with Weight as a factor (4 levels) on each of these features show that trajectory amplitude does not vary among the different lifting actions (horizontal amplitude: *F*(3, 6) = 0.71, *p* = 0.58, *η*_*p*_^*2*^ = 0.26; vertical amplitude: *F*(3,6) = 2.73, *p* = 0.14, *η*_*p*_^*2*^ = 0.58). Action duration and the maximum of the lifting speed modulus instead differ significantly (*F*(3, 6) = 16.13, *p* = 0.003, *η*_*p*_^*2*^ = 0.89 and *F*(3, 6) = 49.5, *p* < 0.001, *η*_*p*_^*2*^ = 0.96 respectively). Bonferroni post-hoc tests indicate that the significant differences are between the two smallest and the two largest weights.

**Table 1:** Features of the lifting actions of the human demonstrator.

Lifting properties	100g	200g	300g	400g
Duration [s]	1.4 ± 0.1	1.5 ± 0.1	2.1 ± 0.3	2.2± 0.2
Amplitude (lateral) [cm]	25.1 ± 0.4	25.3 ± 0.5	25.7 ± 0.3	25.2 ± 0.6
Amplitude (vertical) [cm]	13.2 ± 0.5	14.1 ± 0.3	14.0± 0.4	13.7± 0.4
Max Vel (modulus) [cm/s]	44.4 ± 0.8	41.9 ± 3.0	34.2 ± 2.2	29.2 ± 0.2

The table reports the duration of the lifting movement, computed as the time between lifting onset and termination, identified by applying a velocity threshold of about 1cm/s to the vertical velocity; movement amplitude, computed as the horizontal and vertical extension of the motion, respectively; and hand peak velocity, i.e., the maximum speed of the lifting movement.

### Data analysis

For all subjects, we computed the mean estimate for each weight by averaging all the responses for a given stimulus. Moreover, we computed the mean weight estimate across the whole session by averaging all judgments to determine the sign of the error in judgments (over- or underestimation). To evaluate whether performance changed with age in terms of accuracy or precision, we partitioned the total error of the estimates into two, as in [25]. For each weight presentation, we computed the bias (i.e., the absolute difference between the estimate and the actual weight) to represent the accuracy of the reproduction, and the variability of the estimates (i.e., the standard deviation of the different estimates for the same weight). This is inversely related to the precision of the judgment. In detail, the bias for each *i*-th weight value of the stimulus corresponded to the difference between the average estimated weight $\left({\bar{E}}_{i}\right)$ and the sample weight (*S_i_*), normalized by the average stimulus weight in the experiment $\left(\bar{S}=250g\right)$:

$$BIAS_{i}=\frac{\left|{\bar{E}}_{i}-S_{i}\right|}{\bar{S}}$$ (1)

The variability resulted from the standard deviation of the *N* estimates *E_i_* for each *i*-th stimulus weight, again normalized by the average stimulus weight in the experiment $\left(\bar{S}\right)$:

$$SD_{i}=\frac{\sqrt{VAR_{i}}}{\bar{S}}=\frac{\sqrt{\sum {\left(E_{i}-{\bar{E}}_{i}\right)}^{2}/N}}{\bar{S}}$$ (2)

From these values we computed two summary statistics for each participant: BIAS, the root mean square of BIAS_i_; and SD, the square root of the average of VAR_i_ across all weights.

The total error for each participant was then computed as the Pythagorean sum of BIAS and SD:

$$RMSE=\sqrt{BIAS^{2}+SD^{2}}$$ (3)

The current study assessed developmental changes in all performance parameters through one-way ANOVAs with Age as factor, followed by Bonferroni post-hoc tests. Judging weight from lifting observation is thought to involve the observer’s motor system, so we accordingly evaluated whether changes in performance during childhood could correlate more strongly to a measure of the body structure than chronological age. Among the possible proxy of physical size, we considered children’s height, which undergoes a substantial change between 6 and 10 years of age. We therefore ran a multiple regression analysis over the whole children sample (6- to 10-year-olds), with Total Error (measured as RMSE, Eq 3) as the dependent variable and Height and Age in Months as predictors. Also, we ran ANCOVAs on RMS, precision and accuracy with height as covariate. Finally, we ran a Two-Ways Mixed Model ANOVA on the estimates to assess whether participants could reliably discriminate among the different weights and whether their estimates changed with age, with Age as a between factor (4 levels– 6YO, 8YO, 10YO and 32YO) and Weight as the within factor (4 levels– 100g, 200g, 300g, 400g) followed by Bonferroni post-hoc tests. Statistical significance was considered reached for p-values < 0.05.

## Results

**Fig 2:**
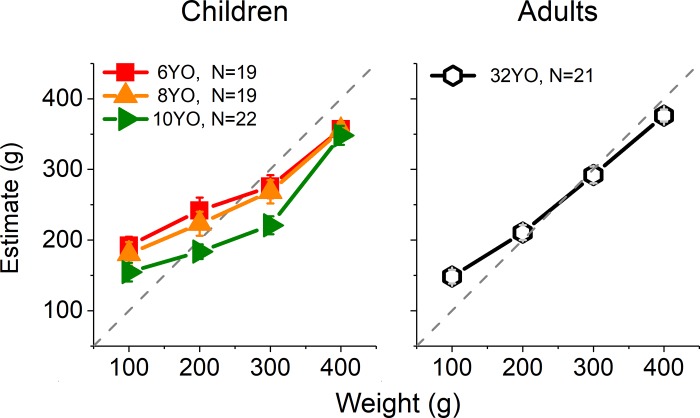
Mean estimated weight from lifting observation at different ages. Error bars represent standard errors of the mean (SEM). The identity lines (dashed) represent perfect estimation.

**Fig 3:**
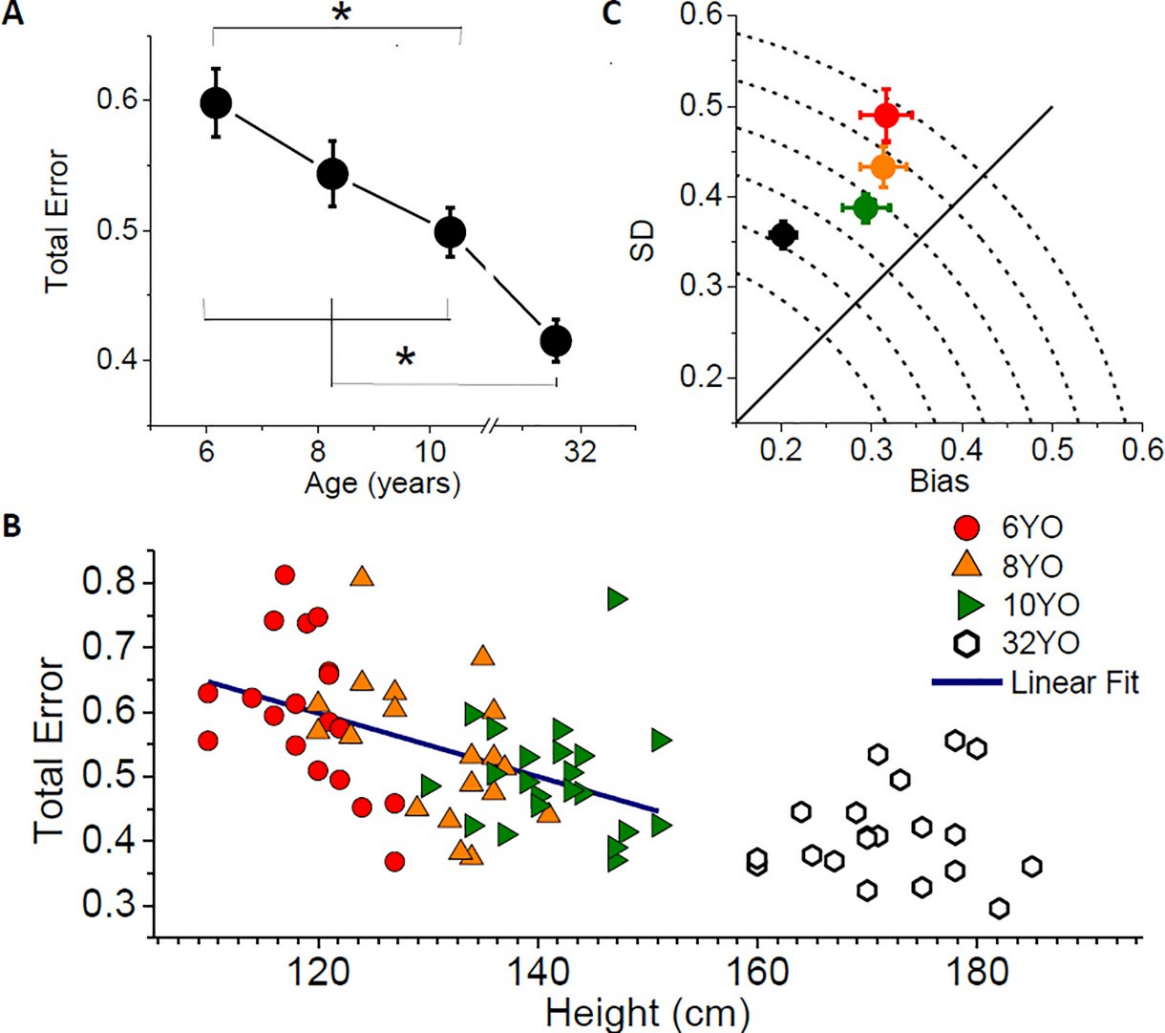
Performance in weight judgments. A) Average Total Error (RMSE, Eq 3) of the estimates, normalized by mean stimulus weight (250g). Error bars represent SEM. Stars indicate significant difference in a one-way ANOVA followed by Bonferroni post-hoc tests. B) Individual RMSE as a function of subject’s height. Different symbols represent different age groups. The blue line represents the linear fit of the children data (Adj. R^2^ = 0.22; p < 0.001). C) Average variability of the estimates (SD, Eq 2) plotted against Bias (Eq 1) for the different age groups. The Total Error (RMSE, Eg 3) is given by the distance from the origin. All variables are normalized by the mean stimulus weight (250g). Error bars represent group SEM.

Fig 2 shows the weight estimates made by the different age groups after observation of a human actor lifting bottles of different weights. The figure makes it evident that, from six years of age, children can discriminate different weights from action observation, with estimates increasing alongside the weights actually lifted. The average error in the estimates (RMSE, normalized by the average stimulus weight, Eq 3) decreased significantly with age, dropping from about 150g at 6 years of age (*M* = 149, *SD* = 24g) to around 100g in adulthood (*M* = 104, *SD* = 18g, see Fig 3A and 3B). A one-way ANOVA on RMSE confirmed that error varied with age (*F*(3,77) = 12.81, *p* < 0.001, *η*_*p*_^*2*^ = 0.33), with a significant drop between 6 and 10 years (Bonferroni post-hoc, *p* < 0.001) and between all children groups and adults (Bonferroni post-hoc tests, 6-year-olds: *p* < 0.001, 8-year-olds: *p* < 0.001, 10-year-olds: *p* = 0.035; no other comparison reached significance). Similar results occur when one removes adults from the analysis (*F*(2,57) = 4.7, *p* = 0.01, *η*_*p*_^*2*^ = 0.14).

To assess whether performance correlated more with changes in physical structure than chronological age, we ran a multiple regression analysis for the entire children sample (6- to 10-year-olds). In the analysis, Total Error (RMSE) was a dependent variable and Height and Age in Months were predictors. The regression was significant (*p* < 0.001, adjusted *R*^2^ = 0.212), and the only variable wherein the estimated coefficient in the linear model was significantly different from 0 was Height (*p* = 0.01; Age: *p* = 0.5). To further validate the role of height as predictor of subjects’ performance, we ran an ANCOVA on Total Error (RMSE) with Age as a factor and Height as the covariate. When controlling for height, results show there is no significant effect of age on total error (*F*(3,76) = 0.29, *p* = 0.832, *η*_*p*_^*2*^ = 0.06),confirming that Height is therefore a better predictor of task performance. Similar results occur when one remove adults from the analysis (*F*(2, 56) = 0.25, *p* = 0.780, *η*_*p*_^*2*^ *=* 0.008).

To assess whether the error decreased during childhood for an increase in the accuracy of the judgments (bias reduction) or in precision (variability reduction) we partitioned the total error into Bias and SD (see Data Analysis and Fig 3C). Absolute accuracy changed significantly in adulthood, ranging around 77g (*SD =* 3g) among the entirety of the children sample and decreasing to 51g (*SD =* 17g) in adults (one-way ANOVA on Bias, *F*(3,77) = 4.98, *p* < 0.01, *η*_*p*_^*2*^ *=* 0.16; Bonferroni post-hoc tests are only significant for comparisons with the adult groups, 6-year-olds: *p* = 0.008, 8-year-olds: *p* = 0.011, 10-year-olds: *p* = 0.044). Conversely, between 6 and 10 years of age, the variability in the judgments decreased sensibly from about 122g (*SD =* 31g) to 97g (*SD =* 18g, *F*(3,77) = 7.77, *p* < 0.01, *η*_*p*_^*2*^ *=* 0.23; significant Bonferroni post-hoc comparisons between 6-year-olds and 10-year-olds, *p* = 0.004, and between 6-year-olds and adults, *p* < 0.001; no other comparison was significant). For all the ages tested, the ratio between the two error components was approximately constant (one-way ANOVA, *F*(3,77) = 1.27, *p* = 0.271, *η*_*p*_^*2*^ *=* 0.05, with SD being on average 1.728 (*SD* = 0.19) times the bias (see the almost constant angle subtending all symbols in Fig 3C)., Considering only the children samples, running the same analyses confirmed these results (accuracy: *F*(2,57) = 0.22, *p* = 0.8, *η*_*p*_^*2*^ *=* 0.001; variability: *F*(2,57) = 5.38, *p* = 0.007, *η*_*p*_^*2*^ *=* 0.16; ratio: *F*(2,57) = 0.74, *p* = 0.48, *η*_*p*_^*2*^ *=* 0.02). To assess the role of height as predictor of subjects’ accuracy and precision, we ran two ANCOVAs on Bias and SD on the whole sample with Age as a factor and Height as the covariate. When controlling for height, results show there is no significant effect of age on Bias (*F*(3, 76) = 0.502, *p =* 0.682, *η*_*p*_^*2*^ = 0.02), nor SD (*F*(3, 76) = 1.22, *p =* 0.307, *η*_*p*_^*2*^ = 0.05).

**Fig 4:**
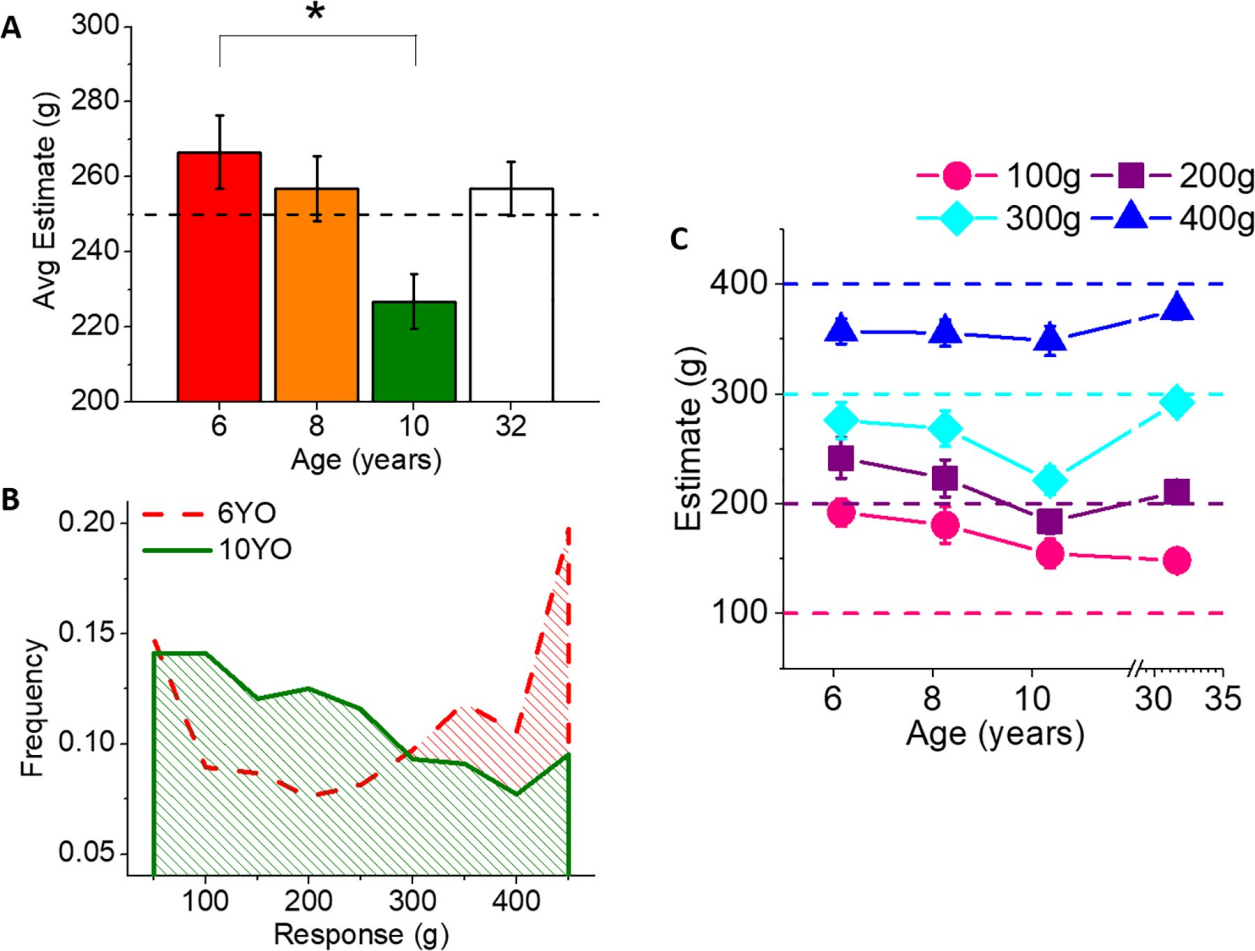
Weight estimates. A) Average estimated weight for the whole experiment by the different age groups. The actual average weight was 250g (dashed horizontal line). Error bars represent SEM. Stars indicate significant difference in a one-way ANOVA followed by Bonferroni post-hoc tests. B) Frequency distribution of subjects’ responses across the 9-point scale, for 6 and 10 YO. C) Average estimates (± SEM) for the different weights presented in the movies, as a function of age. Different symbols represent different stimulus weight.

Although absolute accuracy on average did not change substantially during childhood (see Bias in Fig 3C), Fig 2A indicates that, on average, the sign of the errors (i.e., underestimation/overestimation) was different for the three age groups the current study tested. To investigate this phenomenon further, we computed the average estimate for each participant across the whole experiment. The predicted value for a balanced estimation of the presented weights would have been 250g. Fig 4A shows how such an estimate changes with age. The average estimates vary significantly for the ages tested (one-way ANOVA, *F*(3,77) = 4.612, *p* = 0.005, *η*_*p*_^*2*^ = 0.15), with 10-year-olds values that are significantly smaller than 6-year-olds (Bonferroni post-hoc, *p* = 0.006; no other comparison reached significance). Indeed, while the youngest group produced an estimate of 267g (*SD =* 43g) on average, older children underestimated the average weight (*M =* 227g, *SD* = 34g), an underestimation that disappeared at adult age (*M =* 257g, *SD =* 34g). The differences are still significant when we exclude adults from the ANOVA (*F*(2,57) = 6.15, *p* = 0.004, *η*_*p*_^*2*^ = 0.18) and when testing the whole sample controlling for height (ANCOVA, *F*(3,76) = 4.53, *p =* 0.006, *η*_*p*_^*2*^ = 0.15). This result is confirmed by checking the frequency of varying responses (Fig 4B): 10-year-olds showed a bias toward the lighter bottles, in contrast to 6-year-olds, who demonstrated the opposite bias.

A more detailed analysis of the average estimates for the different weights in Fig 4C shows a trend of participants overestimating light weights and underestimating heavy ones at all tested ages. This tendency seems to be stronger in children compared to adults. More precisely, for 100g and 400g, participants of all ages significantly overestimated and underestimated the actual object mass (one-sample t-tests, all *p*’s < 0.001 for 100g and all p’s < 0.002 for 400g). For the 200g bottle, the estimate differed substantially from 200g but only for the youngest age group *(t*(18) = 2.24, *p* = 0.038, *d* = 0.51), while for the 300g object, 10-year-olds were the only ones significantly underestimating object weight (*t*(21) = -6.25, *p* < 0.001, *d* = 1.33). When comparing the average absolute error for each of the 4 weights separately, one can find a significant difference for 100g (One-Way ANOVA, *F*(3,77) = 2.83, *p* = 0.044, *η*_*p*_^*2*^ *=* 0.099) and for 200g (One-Way ANOVA *F*(3,77) = 3.61, *p* = 0.017, *η*_*p*_^*2*^ *=* 0.12). Adults outperform the youngest age group in the estimate of 100g (Bonferroni post hoc one-tailed t-test, *p* = 0.044) and 10-year-olds outperformed the youngest age group in the 200g estimate (Bonferroni post hoc one-tailed t-test, *p* = 0.021). No significant differences emerge for the two heavier masses. Moreover, in the current study’s sample, all children could distinguish the heaviest weight (400g) from others, while adults could correctly discriminate all four different weights. This is confirmed by a Mixed-Model ANOVA (between factor: Age, within factor: Weight) showing that both factors have a significant effect (Age: *F*(3,77) = 4.612, *p* = 0.004, *η*_*p*_^*2*^ = 0.15; Weight: *F*(3,231) = 182.4, *p* < 0.001, *η*_*p*_^*2*^ = 0.70) and interaction (*F*(9,231) = 2.16, *p* = 0.026, *η*_*p*_^*2*^ = 0.08). Concerning all children, the following Bonferroni post-hoc comparisons confirm that the estimates of 400g are different from those for other weights (all *p*’s < 0.001) and the estimate for 100g differs significantly from that for 300g (all *p*’s < 0.001). For adults the estimates for all four weights differed significantly (all *p*’s < 0.03). If adults are excluded from the analysis, the Mixed-Model ANOVA still shows a significant effect of both Age and Weight (*F*(2,57) = 6.15, p = 0.004, *η*_*p*_^*2*^ = 0.18; *F*(3,171) = 99.6, *p* < 0.001, *η*_*p*_^*2*^ = 0.64, respectively) and no interaction (*F*(6,171) = 0.87, *p* = 0.52, *η*_*p*_^*2*^ = 0.03). This suggests that estimates change significantly among the three different age groups, but the relation between weight and estimates remains similar across the three young ages that the current study tested.

## Discussion

In this study we investigated the development of the ability in childhood to judge, through observation, weight being lifted. Our results indicate that children at the age of 6 can reliably discriminate among slightly different weights being lifted by someone else. As soon as children can recognize small differences in object mass through direct lifting–an ability that most master around 6 years of age for a weight range similar to the one tested here [22]–they can also detect small differences in mass lifted by others. This skill improves significantly during childhood, indicated first by a decrease in the variability of judgments and later by an increase in absolute accuracy (Fig 3C). However, even by 10 years of age, children show different performance from adults, specifically by exhibiting a consistent underestimation of lifted weights.

To understand which processes intervene to delay acquisition of adult-like performance in this weight reading task, it may be useful to consider which neural mechanisms are involved. One hypothesis is that this ability contributes not only visual information but also the activation of the motor system of the observer [6], as suggested by the mirror neuron theory [26]. According to this hypothesis, observers simulate observed actions in their own motor repertoire, in order to understand them [27]. For instance, Hamilton and colleagues demonstrated that occupying the motor system by lifting a box tends to bias perceptual judgments of the weight of an object that another person has lifted [28,29]. Moreover, observing motion kinematics modulates the activation of the observer’s own motor system, reflecting both the muscle used in the observed motion and the force produced in a particular muscle [30,31]. It also reveals whether the action was performed with the intent to deceive about the actual object weight [3,32]. Finally, studies indicate that the judgment of weight from human action is selectively impaired when the activity of the Inferior Frontal Gyrus (IFG) [33] is disrupted with repetitive transcranial magnetic stimulation (TMS). The IFG is the frontal node of parietofrontal mirror circuit, which is strongly reciprocally connected to the primary motor area [34]. Importantly, the same disruption of IFG does not impair weight judgment when observing an event not caused by a human action (e.g. a bouncing ball), which proves IFG is activated in tasks requiring (also unconsciously) simulation of the observed action with a detailed motoric representation of its kinematics. In sum, lifted weight estimation involves the activation of the observer’s motor system and motor models.

We speculate that the progressive improvement we have observed in weight judgment until 10 years of age may reflect the development of children’s motor control. Around that age, the development of force regulation reaches maturity, at least in simple force control tasks (such as producing a fixed force with one finger), although further increases in maximum produced force are present until adulthood [35]. Considering more complex exercises, such as grasping to lift or reaching with the whole arm, studies report a clear decrease in the variability of the motion until late childhood. Indeed, variability in grip force rate curves decreases until 8 years of age [36] while movement variability in reaching diminishes significantly until 10–11 years of age [37]. Moreover, children master the task of lifting a series of objects of increasing weights in an optimal way at no earlier than 9 years of age, as assessed from the analysis of the amplitude of the kinematic parameters and the electromyographic data of the motion [38].

Moreover, the maturation of an accurate representation of children’s body dynamics remains incomplete until least 10 years of age [39]. In a task where children 6 to 10 years of age had to adapt to a damping external force during reaching, all young participants exhibited more pronounced aftereffects than adults, demonstrating that imprecise representation of arm dynamics persists until late childhood. Spectral density analyses of the force signal in a finger force production task performed by children of different ages have revealed evidence of an ongoing change toward motor control based on internal representations rather than on feedback loops. Smits-Engelsman and colleagues (2003) have shown that the relative power in the lowest frequencies bands—reflecting the use of visual and proprioceptive feedback in control [40]—decreased linearly with age until humans reach 11–12 years of age. Conversely, power in the 10-Hz range, which is commonly taken as evidence for the need to use feed forward strategies [41], showed an increasing tendency that continued even until adulthood.

In this perspective, our findings suggest that as children mature the internal representation of their own motor system, this also produces an increase in their performance interpreting others’ actions. Possible maturational aspects in cognitive abilities could have contributed to the improvement of weight estimation abilities from childhood to adulthood. Beyond these speculations, our current results clearly demonstrate a long developmental trend in weight reading, highlighting how not all aspects of action perception have already developed during early childhood.

Children’s stature was a more reliable predictor of their abilities in terms of reading others’ actions than their chronological age. Authors have suggested that body height is a parameter more directly related to the growth of the child than his age, considering it is more tightly correlated with the maturation of signal transmission in growing muscles [40] as well as with the increase in the maximum voluntary contraction of the finger muscles [35]. The correspondence between stature and maturation occurs in the entire age range of childhood until 20 years of age, but it is particularly tight around 5–7 years of age. Weight evaluation during action observation may depend on activation of the observer’s motor system [42], which undergoes substantial development during childhood owing to physical growth of the body [43]. A measure such as height could therefore represent a more direct estimate of ongoing child maturation, at the same time explaining its higher power at predicting a child’s skills at judging mass from action observation.

An interesting question when analyzing the results of our work pertains to which kinematic properties of the presented videos participants used for making their weight judgments. While the spatial properties of the lifting actions were almost constant for all the tested masses, the temporal and spatio-temporal features of the movements varied systematically with weight (see Table 1). Indeed, an increase in duration of about 57% and a decrease in absolute velocity of about 52% between 100g to 400g characterized the movies used as stimuli. We therefore suggest that participants exploited one or both of these variables while judging weight. This conclusion is consistent with previous findings on adults, indicating that lifting velocity and duration represent important cues for judging weight [6,44]. They also allow for weight discrimination even when a humanoid robotic actor minimizes the contribution of other visual information [7,45]. However, the validity of this assumption requires evidence that children can discriminate movements based on velocity and/or duration. Both visual speed and duration discrimination remain under development in the age range tested in the current study [46,47]. Nonetheless, already by 5 years of age children can reliably distinguish 30% differences in stimulus duration for static visual stimuli lasting around 1s [47]. They can distinguish about 44% differences in stimulus speed for sinusoidal gratings moving at an average speed of about 6°/s (approximately corresponding to 5cm/s) [48]. Considering that the sensitivity for faster speeds develops earlier than for slower speeds [46], one may expect young children’s speed sensitivity to be even higher for average speeds in the range of those that we used in our experiment. Moreover, at least in adults, time perception of visual motion is more precise and accurate for biological motion than for different kinematics [49]. This suggests that sensitivity to the kinematic cues in our movie stimuli could be even higher than that measured for different artificial stimuli. Overall, it seems plausible that participants in our study could use velocity and duration cues in their weight judgments. Future research is needed to verify this claim and assess the relative importance of different kinematics cues and the potential influence of other sources of information, such as the hand contraction state revealed by the whitening and stretching of the skin during force production [30].

Another observation derived from our data is that, at all the ages tested, participants tended to overestimate lighter weights and underestimate heavier ones. This phenomenon, visible also in adults’ data, is even more accentuated in younger groups (Fig 4C). A similar result is consistent with previous studies with regards to the perception of lifted weight among adults (e.g., [50]) and reflects a “contraction effect” [51] or central tendency–that is, judgments of almost all quantities tend to gravitate toward their mean magnitude. Recent evidence demonstrates that this effect is more pronounced when judgment is more imprecise, reflecting a strategy of error minimization [52]. This effect is already present during childhood from at least 7 years of age [53]. We suggest that our results reflect a central tendency effect that is particularly enhanced by task complexity. Indeed, in our experiment we did not explicitly present the magnitude to judge, but instead implicitly encoded it in the kinematics of the lifting, potentially increasing judgment imprecision.

Analysing participants’ estimates has highlighted that accuracy does not improve significantly in childhood, although performance in weight reading increases progressively with age.

These findings point toward two different phenomena. The first is an increase in the precision of weight estimates, perhaps associated with noise reduction in motor control and improvements in precision of force tuning that occur in this age span. The second phenomenon is the estimation of the weight of the object (or of the effort of the lifter) remaining offset at 10 years of age, which suggests that the mapping between oneself and the actor is still inaccurate at that age. These phenomena may result from the physical differences between the body structure of the children and the actor. Indeed, children’s height in the current study was approximately 15cm to about 60cm smaller than that of the actor. At the same time, height ended up being a more reliable predictor of participants’ performance than their age.

To evaluate if height difference from the model could explain differences in performance, we assessed whether errors changed as a function of this parameter in the adult sample, where the other developmental processes are already completed. In the current study we found no evidence in favor of this hypothesis. In particular, the linear fit of Total Error with respect to difference in height between actor and observer for the adult sample alone was non-significant (Adj. R^2 ≅^ 0; p = 0.67).

Additionally, if the “calibration” of the estimates were driven by height difference alone, we might have expected a progressive trend of under- or overestimation of the weights across different age groups. This is because the difference in height increased progressively with the younger ages. Conversely, the sign of average weight estimates follows a non-linear trend, with 10-year-olds exhibiting a substantial underestimation of the weight lifted by other people. Such underestimation is not present in younger children or adults, recalling a non-linear U-shaped development [54]. This phenomenon is similar to the pattern of development for size perception. When judging the dimension of a faraway object, young participants significantly underestimate its dimension up until 14 years of age, while adults show the opposite bias [55,56]. Previous results in adults have indicated that, in weight judgment tasks, subjects are more sensitive to perceived effort than to actual object mass [8]. As an alternative explanation, we propose that underestimation could result from incorrect mapping between the force of the actor and the child’s own, leading to overestimation of the adult actor’s force. Children around 11 and 13 (for boys and girls, respectively) are undergoing substantial growth and approaching the peak in their velocity of increase in stature [43]. They may encounter difficulties in appropriately calibrating their force due to the rapidly changing properties of their bodies. The incorrect assumption that a similar speed in growth would be maintained at a later age would induce an overestimation of the force of an adult, leading to an underestimation of lifted mass. If this hypothesis is correct, we might expect to find even stronger underestimation at later ages (12–13 years of age), considering that growth speed increases even further.

However, in the current study we cannot provide conclusive evidence about a U-shape development, since we lack participants in the age range in which the reversal of the trend might have occurred. Moreover the procedures for children and adults were slightly different–e.g., in the number of trials observed–and this might have contributed to the observed differences. Future studies spanning a larger age range will be needed to address this question.

In summary, the ability of children to perceive detailed aspects of others’ actions follows a long developmental trend tightly linked to one’s physical maturation. The capability of discriminating slightly different weights from lifting observation is already present by 6 years of age. The age traditionally coincides with the completion of the acquisition of fundamental movement skills, including locomotion, postural control and object manipulation (the fundamental movement phase, [57]). Moreover, this ability increases progressively in childhood, wherein child’s height is a more reliable predictor of performances than chronological age. However, humans do not reach adult-like performance by 10 year of age. Future research will determine whether a reduced difference between the observer’s body and that of the actor–as in the observation of another child’s action–may meet with higher judgment accuracy among younger children.
